# A Rare Entity: Adult Asymptomatic Giant Vallecular Cyst

**DOI:** 10.1155/2015/723420

**Published:** 2015-11-24

**Authors:** Mümtaz Taner Torun, Ender Seçkin, Ümit Tuncel, Caner Kılıç, Özalkan Özkan

**Affiliations:** ^1^Bandırma State Hospital, Ear Nose and Throat Surgery Clinic, Bandırma, 10200 Balıkesir, Turkey; ^2^Erzincan University, Ear Nose and Throat Surgery Clinic, 24100 Erzincan, Turkey; ^3^Ankara Oncology Training and Research Hospital, Ear Nose and Throat Surgery Clinic, Yenimahalle, 06200 Ankara, Turkey

## Abstract

*Background*. Cysts in the larynx are rare and generally asymptomatic. However, large cysts in adults can be symptomatic. If they are symptomatic, they typically present with respiratory and feeding difficulties. They are usually benign in terms of pathology. Several surgical techniques may be used for treatment.* Case Report*. A 56-year-old man presented to our clinic with hoarseness. Routine laryngeal examination revealed a giant mass and the larynx could not be visualized. At magnetic resonance imaging (MRI), a cystic mass originating from the vallecula was detected. There was no pathology at the glottic level. We planned tracheotomy for the airway and endoscopic surgery for excision. The mass was excised using CO_2_ laser and was reported as benign.* Conclusion*. An asymptomatic vallecular cyst may cause difficult intubation in any operation. It may also cause respiratory or other complications. Airway management should be led by an ear, nose, and throat surgeon, since tracheotomy may be required. Endoscopic excision with CO_2_ laser is a good choice for treatment in elective cases. In this report, we discuss the diagnosis and treatment of a patient with an asymptomatic giant vallecular cyst.

## 1. Introduction

Vallecula cysts (VCs) arise due to obstruction of the mucous gland duct. Ductal cysts are usually small, approximately 1–5 mm in diameter. Adult VCs are frequently asymptomatic. When seen in adults, possible symptoms include globus, voice changes, dysphagia, hoarseness, and airway obstruction. The lesion is usually fluctuant and some mucoceles may appear at palpation. VCs are benign in pathology and are not associated with other anomalies or syndromes [1]. They can become infected and this can lead to acute epiglottitis with or without abscess formation and this situation may be associated with life-threatening acute airway obstruction [2]. Giant VCs have been observed at the time of induction of general anesthesia. It can result in difficulties in endotracheal intubation. Tracheotomy is sometimes necessary for airway management. Surgical procedures include deroofing, marsupialization, excision with snare, and laser vaporization or excision [3, 4]. We describe a case of a giant, asymptomatic VC operated with CO_2_ laser endoscopically.

## 2. Case Report

The patient gave signed informed consent.

**Figure 1:**
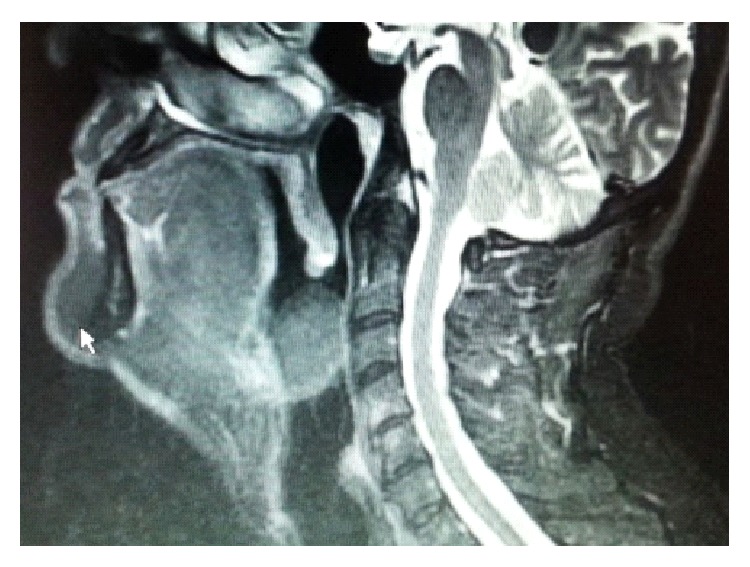
A 4 × 3 cm cystic mass filling the epiglottic vallecula and obstructing the airway.

**Figure 2:**
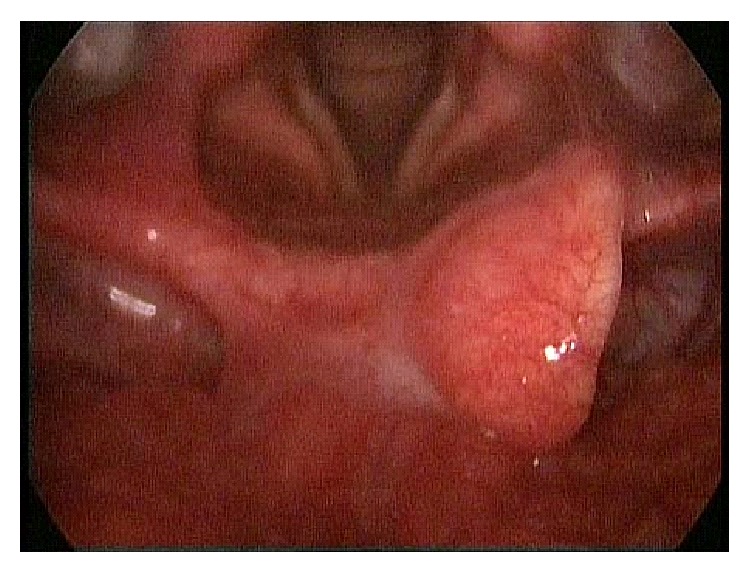
Appearance at 6 months postoperatively.

A 56-year-old man presented to our clinic with hoarseness. Routine ear, nose, and throat clinical examination revealed a translucent, cystic, smooth-surfaced mass displacing the epiglottis posteriorly and completely occluding the laryngeal inlet. No lymphadenopathy was observed on the neck. Other physical examinations were normal. Magnetic resonance imaging (MRI) revealed a 4 × 3 cm cystic mass, originating from the epiglottic vallecula and filling the airway (Figure 1). No sign of malignancy, such as infiltration, was observed at MRI. No edema or inflammation was detected. Thyroid screening was performed and reported as normal. An elective surgery was planned. After the consultation with anesthesiologists, we decided to perform tracheotomy under local anesthesia. General anesthesia was administered subsequently and the cyst was visualised endoscopically. Thirty degree and 70°, 4 mm and rigid fiberoptic telescopes (Karl Storz, Tuttlingen, Germany) were used for visualization during surgery. The content of the cyst was aspirated partially and its origin was identified. The cyst destructed the right side of the epiglottis due to its pressure. It was removed from the vallecula using a CO_2_ laser with endoscopy. Hemostasis was also achieved with CO_2_ laser. The tracheotomy was closed after the operation. The patient was observed overnight and discharged without complication the following day. Voice quality was normal and the patient was fed without difficulty. Even though epiglottis was traumatized, this did not lead to aspiration. No recurrence was observed at 6 months postoperatively (Figure 2).

## 3. Discussion

Laryngeal cysts are classified as ductal and saccular cysts. Ductal cysts result from obstruction and retention of mucus in the collecting ducts of the submucosal glands. Saccular cysts arise from the saccule and extend to the ventricle. Ductal cysts are the most common form of laryngeal cysts, comprising 75% of cases [5]. VCs are generally seen on the lingual surface of the epiglottis [6]. They may present at any age with sporadic occurrence. Their prevalence and incidence are not precisely known. Cases usually appear as case reports in the current literature. They are usually asymptomatic. Stridor, cough, dysphonia, foreign body sensation, hoarseness, and dysphagia are some possible symptoms. Infection of the cyst may spread to the surrounding structures and cause edema and inflammation. Indirect, direct, or flexible laryngoscopies are usually performed to diagnose VCs. Thyroid screening was also performed in our case to rule out lingual thyroid and thyroglossal cyst.

Patients with severe respiratory distress or near total obstruction of the larynx require immediate intervention. Needle cricothyrotomy or emergency cricothyrotomy with a trocar is the immediate management options [7]. Aspiration of cyst contents is possible but not in mucoid cysts. Cyst rupture also creates difficulties in visualizing the vocal cords and an aspiration may occur. Inhalational induction with orotracheal intubation is described as the method of choice in pediatric patients. Open, endoscopic (needle aspiration, marsupialization, and laser ablation), or combined procedures can be used for treatment. An incision scar, increased anesthesia time, a risk of superior laryngeal nerve injury, and long hospitalization are the main disadvantages of open procedures. Limited exposure, difficulty in bleeding management, the need for adequate equipment, high costs, and the risk of thermal or airway injury are the principal disadvantages of endoscopic procedures. We did not experience any of these in our case. No incision scar and shorter operation time and recuperation are the main advantages of the endoscopic approaches.

Tracheotomy can be performed before anesthesia induction to provide a more controlled airway. Using CO_2_ laser with endoscopy is a good treatment option, as in our case. CO_2_ laser has ablative and hemostatic properties and also permits control over depth of penetration into the tissue [8]. The employment of rigid telescopes along with a flexible laser fiber system allows excellent visualization. Recurrences are rare in total excisions. Surgery was completed without complication in our case and no recurrence was observed at 6th month follow-up.

In conclusion, giant VC is a rare entity, especially in adults. Excision with endoscopic CO_2_ laser may allow shorter anesthesia time and avoids the risk of superior laryngeal nerve injury compared with the open approach. It is important to manage the airway in these cases. This rare entity should be remembered in cases of difficult intubation.
